# A descriptive study of smear negative pulmonary tuberculosis in a high HIV burden patient’s population in North Central Nigeria

**DOI:** 10.1371/journal.pone.0238007

**Published:** 2020-09-01

**Authors:** Abdulwasiu Bolaji Tiamiyu, Garba Iliyasu, Farouq Muhammad Dayyab, Zaiyad Garba Habib, Sirajo Haliru Tambuwal, Ayobami Olawale Animashaun, Habibu Galadanci, Sunday A. Bwala, Lovett Lawson, Abdulrazaq Garba Habib

**Affiliations:** 1 Infectious and Tropical Diseases Unit, Department of Medicine, Aminu Kano Teaching Hospital, Kano, Nigeria; 2 Infectious and Tropical Diseases Unit, Department of Medicine, College of Health Sciences, Bayero University Kano, Kano, Nigeria; 3 Lafiya Group of Hospitals, Ibadan, Nigeria; 4 Department of Medicine, National Hospital, Abuja, Nigeria; 5 Zankli Medical Center, Abuja, Nigeria; The University of Georgia, UNITED STATES

## Abstract

Tuberculosis (TB) is a serious disease of public health concern, mainly in low- and middle-income countries. Most of these countries have challenges in diagnosis and treatment of TB in people with smear-negative pulmonary tuberculosis (SNPTB), which remains a significant public health challenge because of the global burden of the disease. We evaluated the epidemiology and clinical presentation of SNPTB in a cohort of patients with high HIV burden. The study was a cross-sectional study among patients with SNPTB in four major hospitals that care for TB/HIV patients in north-central Nigeria. All patients 18 years and above who were newly diagnosed as SNPTB, or patients with SNPTB who had not taken TB drugs for up to 2 weeks irrespective of their HIV status were recruited. Demographic data (sex, age), smoking status, and medical history (clinical form of TB, symptoms at admission, diagnostic methods, presence of comorbidities, prior TB treatment) were obtained using a semi-structured questionnaire. Detailed clinical examination was also done on all the study subjects. Baseline results of packed cell volume, HIV test and sputum acid fast bacilli done during TB screening were retrieved from the patients’ case notes and recorded. Also, the base line Chest X-ray films taken during TB screening were reviewed and reported by two radiologists blinded to each other’s reports. The Xpert MTB/RIF tests and sputum culture (using LJ medium) were done in a TB reference laboratory. A total of 150 patients with SNPTB were studied. Majority of the patients were female 93 (62%). The median age of the patients was 36.5 years with greater percentage of the patients within the ages of 25–44 years 92 (61.3%). Twenty-two (14.7%) of the patients had previous TB treatment. History of cigarette smoking was obtained in only 7(4.7%) of the patients while 82 (64.1%) were HIV positive. All the patients had a history of cough for over a period of at least three weeks, while, 27 (18%) reported having hemoptysis. About 87 (58%) had fever and 110 (73.7%) had anemia, while weight loss and night sweat were reported in 98(65.3%) and 82 (54.7%) of the patients respectively. Chest x rays were reported as typical of TB in only 24 (16%) of the patients. Of the 150 sputa sample analyzed, 21/150 (14.0%) and 22/150 (14.7%) where Gene Xpert and sputum culture positive respectively. The sensitivity and specificity of Gene Xpert assay were 81.8% (18/22; 95% CI 61.5 to 92.7%) and 97.4% (112/115; 95% CI 92.6 to 99.1%), respectively. The study found cough, fever and anemia to be the commonest presentation in patient with SNPTB in a high HIV burden patient’s population. There is also relatively high culture positivity among the patients. This underscores the need to expand the facilities for culture and confirmation in TB centers across the country.

## Introduction

Tuberculosis (TB) is a major Public Health issue worldwide, particularly in low- and middle-income countries [1]. An estimated one-quarter of the world’s population is infected with *Mycobacterium tuberculosis*, with the highest prevalence of the disease found in sub-Saharan Africa and Asia [2]. Infection with HIV is one of the major risk factors for TB and more than half of TB patients live in countries ravaged by Human Immuno-Deficiency Virus/Acquired Immune Deficiency Syndrome (HIV/ AIDS) [3]. Nigeria is ranked as one of the 30 high TB burden countries in the world and it has incidence rate of 219 per 100,000 populations with an estimated 12% of the cases co- infected with HIV [4]. Extra Pulmonary TB (EPTB) is very common among people living with HIV (PLWH) in sub Saharan Africa and it accounts for high mortality and loss to follow up [5, 6]. The prevalence of EPTB among notified TB cases in Africa region in 2017 was 16%, next to the highest Eastern Mediterranean region (24%) [7].

Smear negative pulmonary tuberculosis (SNPTB) remain a major public health challenge because of the global burden of the disease. This is because of increased incidence, under-recognition and under diagnosis; and poor management practices [8]. It is estimated that SNPTB accounts for 30 to 60% of cases of pulmonary tuberculosis (PTB) and causes significant morbidity and mortality, particularly in HIV-prevalent settings [9]. In a study done in Kano (Northwestern Nigeria) by Iliyasu *et al*, the proportion of sputum-negative HIV patients was 82.5% [10], while a previous study in Maiduguri (Northeastern Nigeria) by Moses *et al* reported a figure of 53% among TB/HIV co- infected patients [11].

Mycobacterium tuberculosis is routinely diagnosed by sputum smear microscopy in most resource limited settings; this test has a low sensitivity, and even lower in HIV positive patients [12]. This is because of the paucibacillary nature of SNPTB and its inability to form a cavitatory lesion. In developing countries, the majority of SNPTB cases have been treated only on the basis of clinical and chest radiographic findings [13].

Despite the burden of SNPTB and HIV in Nigeria, little is known about SNPTB. This study therefore, evaluated the epidemiology and clinical presentation of SNPTB in a cohort of patients with high HIV burden.

## Materials and methods

### Site

The study was a cross-sectional study among patients with SNPTB in four major hospitals that cares for HIV and TB patients in Abuja. It also serves as a referral centers to states in North Central Nigeria. The hospitals were; National Hospital Abuja, Asokoro, Maitama and Wuse- District Hospitals. Abuja, the Federal Capital City of Nigeria has a total land area of 713 square kilometers (km^2^) and a population density of 3,423 persons per square kilometer. Its estimated population is 3.6 million according to the 2016 population estimate [14].

### Diagnosis and management of PTB

As of the time of the study, TB patients in the study sites are managed based on the National TB and Leprosy Control Programme (NTP) guidelines [15]. Any patient with unexplained chronic cough for more than 3 weeks is asked to submit three sputum samples for sputum-smear microscopy for acid-fast bacilli (AFB). If the sputum is AFB-positive the patient is registered as a ‘smear positive’ case, and treated with ‘short-course’ TB treatment. Smear-negative PTB is defined as having at least two sputum specimens negative for AFB, a radiological abnormalities consistent with active PTB, failure to improve after a course of broad-spectrum antibiotics (excluding anti-TB drugs, fluoroquinolones and aminoglycosides) [13].

### Patient recruitment

All patients 18 years and above who were newly diagnosed as SNPTB, or patients with SNPTB who had not taken TB drugs for up to 2 weeks irrespective of their HIV status were recruited. Patients with extra-pulmonary TB were excluded from the study. The patients were recruited between September, 2014 to March, 2015 using convenient sampling method.

Using a standardized semi structured questionnaire, the following information was obtained: demographic data (sex, age), smoking status, and medical history (clinical form of TB, symptoms at admission, diagnostic methods, presence of comorbidities, prior TB treatment). A current smoker was defined as reporting smoking at least 100 cigarettes in their lifetime, and at the time of the survey were smoking at least one day a week. A former smoker was defined as reporting smoking at least 100 cigarettes in their lifetime but who, at the time of the survey, did not smoke at all. Never smoked reported having smoked < 100 cigarettes in their lifetime [16]. Detailed clinical examination was also done on all the study subjects and recorded in the questionnaire.

Baseline results of packed cell volume, HIV test and sputum AFB done during TB screening were retrieved from the patients’ case notes and recorded. Also, the base line Chest X-ray films taken during TB screening were reviewed and reported by two radiologists blinded to each other’s reports. These were recorded in the questionnaire as typical (the presence of nodular, alveolar, or interstitial infiltrates predominantly affecting the zones above the clavicles or upper zones; the presence of cavitation affecting the upper zones or the apical segment of the lower lobe, enlarged hilar nodes, pneumonic lesion, atelectasis, mass lesion, miliary) or atypical for pulmonary tuberculosis [17].

On the spot early morning sputum was collected from the patients who satisfied the inclusion criteria, in a well-ventilated space into a sterile container with a tight cover. Each sputum sample collected was divided into 2 aliquots, for the Xpert MTB/RIF assay and culture, respectively. The collected sputa specimens were transported daily in a cold box to the laboratory maintained at a temperature of about 4^o^c. The Xpert MTB/RIF tests and sputum culture (using Lowenstein-Jensen medium) were done in a Biosafety level 3 TB reference laboratory.

### Ethical approval

The study was approved by the Federal Capital Territory Health Research Ethics Committee, Abuja (protocol approval number: FHREC/2013/01/22/28–06–13). Approval from other relevant ethics committees and informed consent were obtained.

### Statistical analysis

Data was analyzed using Statistical Package for Social Sciences (SPSS) version 21 for windows (SPSS Inc., Chicago, IL, USA). Data collected in the study proforma was entered using numeric codes. Frequency distribution tables of variables were generated. Measures of central tendency and dispersion of quantitative variables as well as proportion for qualitative variables were determined. Chi-square test and Fisher's Exact test as appropriate, were used to test significance of the differences between categorical variables.

## Results

A total of 150 patients with SNPTB were studied. Majority of the patients were female 93 (62%), with female to male ratio of about 2:1. The median age of the patients was 36.5 years with greater percentage of the patients within the ages of 25–44 years 92 (61.3%) (Table 1).

**Table 1:** Socio-demographic characteristics of the patients.

Variables	Number (%)
**Age group**	
<25	15 (10.0)
25–44	92 (61.3)
>45	43 (28.7)
**Sex**	
Male	57 (38.0)
Female	93 (62.0)
**Marital status**	
Unmarried	39 (26.0)
Married	97 (64.7)
Divorced	6 (4.0)
Widowed	8 (5.3)
**Education**	
None	13 (8.6)
Primary	9 (6.0)
Secondary	88 (58.7)
Tertiary	40 (26.7)
**Occupation**	
Employed	101 (67.3)
Not employed	49 (32.7)

Twenty-two (14.7%) of the patients had previous TB treatment out of which majority 16 (72.7%) had completed it. History of cigarette smoking was obtained in only 7(4.7%) of the patients while 82 (64.1%) were HIV positive. All the patients had a history of cough for over a period of at least three weeks, while, 27 (18%) reported having haemoptysis. About 87 (58%) had fever and 110 (73.7%) had anaemia, while weight loss and night sweat were reported in 98(65.3%) and 82 (54.7%) of the patients respectively (Table 2).

**Table 2:** Clinical and laboratory characteristic of the patients.

Variables	Study findings n = 150	Davis et al.[20] n = 72	Campos et al.[28] n = 129
	Number (%)	Number (%)	Number (%)
Age(yr)	36.5*	32.0*	46.6^#^
Male sex	(38.0%)	34(47.2%)	74(57.4%)
Haemoptysis	27(18%)	14(19.4%)	4(3.1%)
Fever	87(58%)	67(93.1%)	59(45.7%)
Weight loss	98(65.3%)	69(95.8%)	69(53.5%)
Night sweat	82(54.7%)	-	39(30.2%)
Contact history	11(7.3%)	-	-
Previous tuberculosis	22(14.7%)	-	20(15.5%)
History of smoking	7(4.7%)	-	47(36.4%)
Anaemia	110(73.3%)	-	-
Adenopathy	21(14.0%)	-	-
*Body Mass Index*			-
Underweight	28(18.7%)	-	-
Normal	69(46.0%)	-	-
Overweight	32(21.3%)	-	-
Obese	21(14.0%)	-	-
HIV Positive	94(62.7%)	14(19.0%)	53(41.1%)
Typical chest x ray	24(16.0%)	56(77.8%)	75 (58.1%)

Chest x rays were reported as typical of TB in only 24 (16%) of the patients, while the rest 126 (84.0%) were reported as atypical. In a subgroup of patients with HIV co-infection only 29 (25.0%) of the chest-x-rays was reported as typical (Table 2). Of the 150 smear (AAFB) negative sputa cultured, 22 (14.7%) and 7(4.7%) grew *Mycobacterium tuberculosis* and *Non-Tuberculous Mycobacterium* (NTM) respectively, giving an overall sputa culture positivity of 29(20.1%). Six (4.0%) of the specimens were contaminated and 115 (76.7%) yielded no growth.

Table 3 shows the association between the clinical and laboratory characteristics with sputum culture results. There was a statistically significant association between positive Gene Xpert MTB/RIF result (p<0.0001) and chest X ray findings consistent with TB (p<0.0001) with positive culture result. However, there was no statistically significant association between HIV status and sputum culture for MTB (p>0.05). In a logistic regression model only positive Gene Xpert results was found to be associated with positive sputum culture [odds ratio (OR) = 284.98, 95% Confidence Interval (CI) = 36.72–2211.68, p<0.001] (Table 4).

**Table 3:** Association of clinical and laboratory characteristics with sputum culture.

Variable	Culture Positive n = 22	Culture Negative n = 114	Unadjusted Odds Ratio [95% CI; *p* value]
Haemoptysis	7(31.8%)	18(15.8)	2.49 [0.89–6.96; (p = 0.08)]
Fever	14 (63.6%)	62(54.4)	1.47 [0.57–3.77; (p = 0.43)]
Weight Loss	20 (90.9%)	70(61.4)	6.29 [1.40–28.22; (p = 0.02)]
Excessive Sweating	12 (54.6%)	65(58.0)	0.87 [0.35–2.18; (p = 0.76)]
Past TB Treatment	1 (4.5%)	17(14.9)	0.27 [0.03–2.16; (p = 0.22)]
History of Smoking	3 (13.6%)	1(0.9)	17.84[1.76–180.61;(p = 0.02)]
BCG Scar	14 (63.6%)	60(52.17)	1.60[0.63–4.12;(p = 0.33)]
Adenopathy	8 (36.4%)	13(11.3)	4.48[1.58–12.72; (p = 0.005)]
Anaemia	18 (85.7%)	77(77.8)	0.58[0.16–2.16; (p = 0.42)]
HIV Positive	13(68.4%)	69(63.3)	1.26[0.44–3.56;(p = 0.67)]
Chest X-ray(Typical)	6 (31.6%)	48(45.3)	0.94[0.17–5.14;(p = 0.94)]
Age >45 years	4(18.18)	33(28.95)	0.55[0.17–1.73;(p = 0.30)]
Gene Xpert	17(77.3)	3(2.61)	158.67[32.63–771.47; (p<0.001)]

**Table 4:** Multivariate logistic regression model.

Variable	Adjusted Odds Ratio [95% CI; *p* value]
History of Smoking	741.95 [12.85–42834.74; (p = 0.001)]
Gene Xpert MTB/RIF	284.98 [36.72–2211.68; (p<0.001)]
Weight Loss	13.91 [0.77–252.20; (p = 0.0075)]
Adenopathy	3.07 [0.35–26.83; (p = 0.310)]

Compared to sputum culture (using Lowenstein-Jensen medium), the sensitivity and specificity of Xpert MTB/RIF assay were 81.8% (18/22; 95% CI 61.5 to 92.7%) and 97.4% (112/115; 95% CI 92.6 to 99.1%), respectively. And the positive predictive and negative predictive values were 85.7% and 96.6%, respectively.

## Discussions

Early recognition of active TB could reduce morbidity and mortality, however, the occurrence and clinical presentations of culture-negative PTB have been insufficiently studied [18]. Our study describes the epidemiology and clinical presentation of SNPTB in a high HIV burden patient population. We found majority of the patients to be female, young and HIV positive. Productive cough, fever and night sweat were the common presentation. Culture was positive in a significant number of the patient, with positive Gene Xpert being the only predictor of culture positivity in a logistic regression.

The female preponderance, the HIV prevalence and the age bracket mostly affected are in agreement with previous studies [4, 19–23]. In countries with low prevalence of HIV, SNPTB was more common in older than younger patients [13]. However, in a high prevalence HIV settings similar to our studied population an even distribution has been reported [24], probably because HIV affects younger age-groups [25]. The clinical presentations in this study were similar to that of another study done in Uganda [20]. These similarities could be because both countries have comparable prevalence of HIV and TB infections [26, 27]. In a cohort of patients with previous culture positivity and low HIV burden, Campos et al, [28] reported figures slightly different from our findings, suggesting the influence of HIV in TB presentation. The low sputum culture positivity in our study also is in support of possibility of significant number of patients who might have been missed diagnosed as SNPTB using the WHO algorithm. It has been suggested that; some AIDS related diseases such as *Pneumocystis carinii* pneumonia (PCP), pulmonary Kaposi’s sarcoma, and Gram-negative bacteraemia presents clinically as SNPTB in HIV patients and may be misdiagnosed as such [29].

As demonstrated previously [30, 31], we found majority of the chest-x-ray patterns to be atypical. This is likely because of the smaller burden of mycobacteria in SNPTB especially in areas with high prevalence of HIV [32]. This make diagnosis of TB even more challenging to clinicians [33].

The culture contamination rate of this study is considerably lower than the 8.8% [34] and 7% [35] reported in other part of Nigeria. This is likely because these studies used a relatively larger study population as well as liquid culture media which has a higher contamination rate [36]. We found higher culture positivity rate compared to the 2.6% reported by Kehinde et al [23], in a retrospective laboratory based study that also involved other specimens apart from sputum. The study design may be responsible for the difference.

The sensitivity of Xpert MTB/RIF in this study was comparable to 82.9% and 82% reported in general population and in PTB-HIV coinfection by Justin et al. and Chang et al. respectively [37, 38]. The specificity of Xpert MTB/RIF in this study was also, comparable to other studies conducted in in general population in high and low TB burden countries; 94% in Zambia [37], 98% in Peru, Azerbaijan and South Africa [38, 39] and 92% in the Netherlands [40].

The findings of the study describe the common presentations in patient with SNPTB as well as high culture positivity among the patients. This underscores the need to expand the facilities for culture and confirmation in TB centers across the country. This will help in reducing false positive and false negative cases using the WHO algorithm, therefore, curtailing unnecessary treatment costs and toxicities as well as reducing the risk of TB transmission. Further similar large scale studies especially in other geographical regions of the country are required to appreciate the burden of TB in sputum smear negative patients. This would enhance policy renewal as it concerns the diagnosis of SNPTB and treatment of MDR-TB.
